# Gallbladder microbiota composition is associated with pancreaticobiliary and gallbladder cancer prognosis

**DOI:** 10.1186/s12866-022-02557-3

**Published:** 2022-05-27

**Authors:** Mari Kirishima, Seiya Yokoyama, Kei Matsuo, Taiji Hamada, Michiko Shimokawa, Toshiaki Akahane, Tomoyuki Sugimoto, Hirohito Tsurumaru, Matsujiro Ishibashi, Yuko Mataki, Takao Ootsuka, Mitsuharu Nomoto, Chihiro Hayashi, Akihiko Horiguchi, Michiyo Higashi, Akihide Tanimoto

**Affiliations:** 1 Department of Pathology, Graduate School of Medical and Dental Sciences, Kagoshima University, Kagoshima Kagoshima, Japan; 2 Department of Data science, Faculty of Data science, Shiga University, Otsu, Shiga Japan; 3 Department of Food Science and Biotechnology, Faculty of Agriculture, Research Field in Agriculture, Kagoshima University, Kagoshima, Kagoshima Japan; 4 Department of Digestive Surgery, Breast and Thyroid Surgery, Graduate School of Medical Sciences, Kagoshima University, Kagoshima, Kagoshima Japan; 5 Department of Gastroenterological Surgery, Fujit a Health University Bantane Hospital, Nagoya, Aichi Japan

**Keywords:** Pancreaticobiliary tract, Gallbladder, Cancer, Bile, Microbiota, Alpha diversity, Beta diversity, Overall survival, Prognosis, Biomarker

## Abstract

**Background:**

The microbial population of the intestinal tract and its relationship to specific diseases has been extensively studied during the past decade. However, reports characterizing the bile microbiota are rare. This study aims to investigate the microbiota composition in patients with pancreaticobiliary cancers and benign diseases by 16S rRNA gene amplicon sequencing and to evaluate its potential value as a biomarker for the cancer of the bile duct, pancreas, and gallbladder.

**Results:**

We enrolled patients who were diagnosed with cancer, cystic lesions, and inflammation of the pancreaticobiliary tract. The study cohort comprised 244 patients. We extracted microbiome-derived DNA from the bile juice in surgically resected gallbladders. The microbiome composition was not significantly different according to lesion position and cancer type in terms of alpha and beta diversity. We found a significant difference in the relative abundance of *Campylobacter*, *Citrobacter*, *Leptotrichia*, *Enterobacter*, *Hungatella*, *Mycolicibacterium*, *Phyllobacterium* and *Sphingomonas* between patients without and with lymph node metastasis.

**Conclusions:**

There was a significant association between the relative abundance of certain microbes and overall survival prognosis. These microbes showed association with good prognosis in cholangiocarcinoma, but with poor prognosis in pancreatic adenocarcinoma, and vice versa. Our findings suggest that pancreaticobiliary tract cancer patients have an altered microbiome composition, which might be a biomarker for distinguishing malignancy.

**Supplementary Information:**

The online version contains supplementary material available at 10.1186/s12866-022-02557-3.

## Background

Adenocarcinoma of the pancreaticobiliary tract and gallbladder has a high rate of mortality despite advanced diagnostic techniques. Surgical resection is the best treatment option for pancreaticobiliary tract cancers; however, the high recurrence rate after surgery significantly affects the disease outcome [1–3]. Furthermore, many patients present with unresectable tumors at the time of diagnosis and have limited chemotherapeutic options [4, 5]. Gallbladder cancer is more common in females and is characterized by rapid progression and early metastasis [6]. Treatment options include surgery, chemotherapy, and radiotherapy [7, 8]. However, gallbladder cancer is also usually diagnosed at an advanced stage due to the lack of early signs and clinical symptoms, as is pancreaticobiliary tract cancer, which limits the selection of therapy and undermines a better prognosis. Therefore, it is crucial to identify an effective biomarker to enable early diagnosis and predict the prognosis of gallbladder and pancreaticobiliary cancer.

The human body is colonized by over 100 trillion symbiotic microorganisms (almost equivalent to the number of cells in a human) and collectively referred to as the microbiota [9, 10]. Due to environmental differences, each site in the body is home to distinct microbial ecosystems [10]. Of them, the most diverse bacterial populations occur in the intestinal tract [11, 12]. The human gut microbiota contributes to host physiologic development and maintenance, including education of the host immune system, nutrient digestion, and defense against colonization by pathogenic microorganisms [13, 14]. The gut microbiota is increasingly considered an important factor associated with both tumor development and the efficacy of anticancer therapies [15, 16].

Bile juice was considered sterile due to the difficulties in accessing biological samples, but recent reports indicate the existence of a microbial ecosystem in people with and without hepatobiliary disorders [17, 18]. Furthermore, a clinical study using metagenomic analysis showed the association between carcinogenesis with liver flukes and microbiota in biliary tract cancers [19]. Other studies have shown that intrapancreatic microbiota may mediate tumor resistance to gemcitabine [20]. Hence, a better understanding of the roles of microbes in the development of hepatobiliary-pancreatic tumors may reveal opportunities to develop new prevention and treatment strategies for patients with hepatobiliary-pancreatic cancers by targeting microbes and the microbiota.

In this study, we performed 16S rRNA gene amplicon sequencing analysis of bile juice collected from resected gallbladders in cases of pancreaticobiliary tract and gallbladder cancers to investigate whether alterations in microbiota composition in the gallbladder affect the patient’s prognosis after surgery. We anticipated that the composition of individual microbiomes might be a novel biomarker to predict the prognosis of pancreaticobiliary tract and gallbladder cancers.

## Results

### Differences of microbiome composition in the gallbladder

**Fig. 1:**
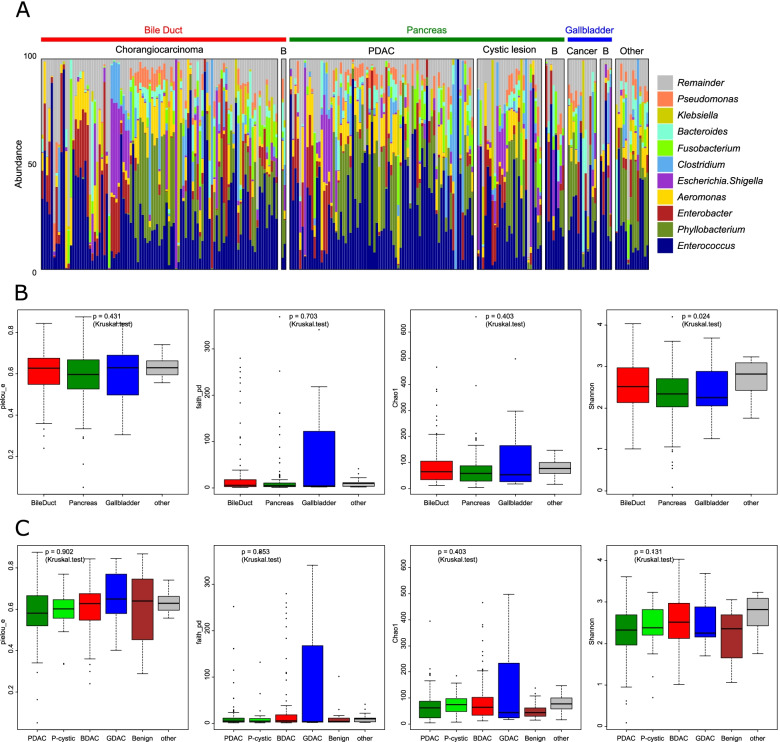
The alpha analysis of microbiota in Bile juice collected from gallbladder. Microbiota alpha diversity in bile collected from resected gallbladders in pancreaticobiliary tract and gallbladder cancers. Microbiome composition analysis at the genus level (**A**). Alpha diversity of Shannon, Chao1, and Pielou evenness indices as well as Faith PD according to the location of the lesion (**B**) or type of lesion (**C**). PDAC, pancreatic ductal adenocarcinoma; BDAC, cholangiocarcinoma; GDAC, gallbladder cancer; P-cystic, pancreas cystic lesion; faith_pd, Faith PD; pielou_e, Pielou evenness index

**Fig. 2:**
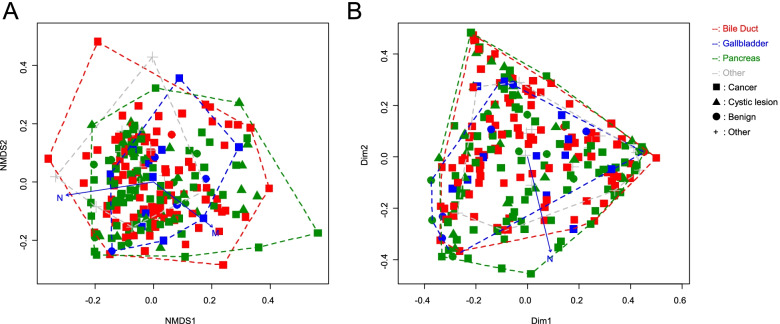
The beta diversity analysis of microbiota in Bile juice collected from gallbladder. Microbiota beta diversity in bile collected from resected gallbladders in pancreaticobiliary tract and gallbladder cancers. The beta diversity analysis at the genus level of 16S rRNA gene amplicon sequencing pattern by NMDS (**A**) and PCoA (**B**) among the lesion locations or lesion type. The type of cancer includes pancreatic ductal adenocarcinoma (PDAC), cholangiocarcinoma (BDAC) and gallbladder cancer (GDAC). The type of cystic is a pancreas cystic lesion. Dim, dimension; N, trends in these variances with the N-score of TNM classification; M, trends in these variances with the M-score of TNM classification; NMDS, Non-metric multidimensional scaling; PCoA, principal coordinate analysis

**Fig. 3:**
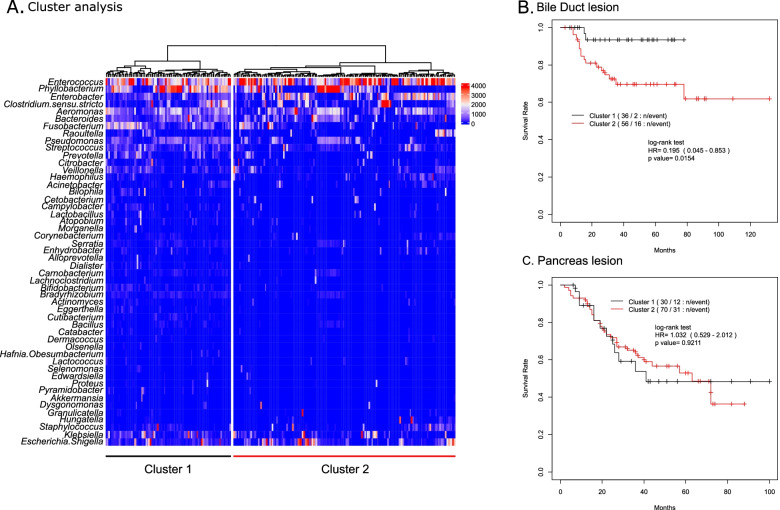
Association between cluster analysis and its prognosis in bile duct lesion and pancreas lesion. Cluster analysis of the relative abundance of microbiota in bile juice at gallbladder. **A** Tree generated by cluster analysis of total sample collected from including bule duct lesion, pancreas lesion and other lesion for the relative abundance (permyriad of total sequences). Cox proportional hazard regression analysis on a comparison between Cluster 1 and Cluster2 **(B)** in bile duct lesion and **(C)** in pancreas lesion. Black line: Cluster 1, red line: Cluster 2

**Fig. 4:**
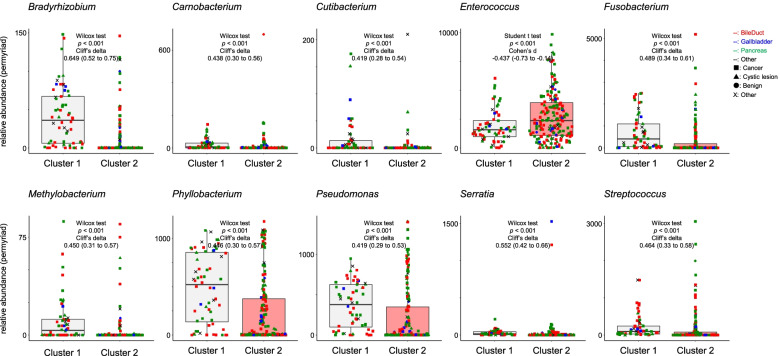
Comparison relative abundance of microbiota between Cluster 1 and Cluster 2. Comparison relative abundance of microbiota between the Cluster 1 and the Cluster 2 on clustering analysis. The color of plot means location of lesion. The red plot, bile duct lesion; the blue plot, gallbladder lesion; the green plot, pancreas lesion; the black plot, other. The plot shape means type of lesion. ■, cancer; ▲, cystic lesion; ●, benign; ×, Other

We isolated the bacterial DNA derived from the bile juice in resected gallbladder samples with pancreaticobiliary tract cancers, gallbladder cancers, pancreas cystic lesions, other cancers, and benign inflammatory lesions. Then, the variable regions (V3–V4) of the 16S rRNA genes were amplified by polymerase chain reaction (PCR). The number of 16S rRNA sequences per bile sample ranged from 10,254 to 342,362. We identified 11,358 ASV (Amplicon Sequence Variants) by subsequent DNA sequencing analysis. Of them, we assigned 19 ASV at the phylum level, 28 ASV at the class level, 61 ASV at the order level, 122 ASV at the family level, and 262 ASV at the genus level (Fig. 1A). Then we assigned ASV at the genus level using BLAST searches (Supplementary Table 1) [21]. There were no differences in the alpha diversity among the lesion locations (Pielou evenness index: *p* = 0.431; Faith PD: *p* = 0.703 and Chao1: *p* = 0.403) without Shannon index (*p* = 0.024) or lesion type (Pielou evenness index: *p* = 0.902; Faith PD: *p* = 0.853; Chao1: *p* = 0.403 and Shannon index: *p* = 0.131) (Fig. 1B, C). Beta diversity of the biliary microbiome was also compared. Non-metric multidimensional scaling (NMDS) of the centered log-ratio-transformed data did not show any distinct clustering, indicating the absence of overall microbiome differences among the types and locations of lesions (Fig. 2A). Similarly, principal coordinate analysis (PCoA) did not show distinct clustering, indicating the absence of overall microbiome differences among the types and locations of lesions (Fig. 2B). However, there was a significant association between trends in these variances and the N-score of the TNM staging system (Supplementary Table 2). Unsupervised hierarchical clustering analysis was performed for relative abundance of microbiota data sets including bile duct lesion, pancreas lesion and other lesion (Fig. 3A). The samples were divided into two clusters according to the clustering results. In bile duct lesion, Cluster 1 showed a significantly better prognosis than Cluster 2 (HR = 0.195, *p* = 0.015; Fig. 3B). However, in pancreas lesion, it has no significant difference prognosis between Cluster 1 and Cluster 2 (HR = 1.032, *p* = 0.921; Fig. 3C). The relative abundance of *Bradyrhizobium*, *Carnobacterium, Cutibacterium, Enterococcus, Fusobacterium, Methylobacterium, Phyllobacterium, Pseudomonas, Serratia* and *Streptococcus* were significant difference between the cluster 1 and cluster 2 (Fig. 4).

### Association between microbial abundance and clinical features

The statistical analysis for the association between microbial individual relative abundance and clinical features is summarized in Table 2. Females showed higher *Escherichia* and *Streptococcus* abundance than males (*p* = 0.007 and *p* = 0.030, respectively). There were significant differences in *Sphingomonas* and *Fusobacterium* between those participants aged over and under 70 years (*p* = 0.011 and *p* = 0.044, respectively). In the cholangiocarcinoma, *Campylobacter*, *Citrobacter* and *Leptotrichia* abundance showed significant increase between Clinical stage with and without lymphnode metastasis (*p* = 0.025, *p* = 0.001 and *p* = 0.007, respectively). In the pancreatic ductal adenocarcinoma, *Enterobacter*, *Hungatella*, *Mycolicibacterium*, *Phyllobacterium* and *Sphingomonas* showed significant difference between with and without lymphonude metastasis (*p* = 0.004, *p* = 0.007, *p* = 0.018, *p* = 0.023 and *p* = 0.058, respectively). Additionally, there were significant differences in the relative abundance of *Schaalia*, *Alloprevotella*, *Bilophila*, *Dialister*, *Eggerthella*, *Selenomonas* and *Streptococcus* between Intraductal papillary mucinous carcinoma (IPMC) and intraductal papillary mucinous neoplasm (IPMN) (*p* = 0.038, *p* = 0.047, *p* = 0.047, *p* = 0.047, *p* = 0.047, *p* = 0.047 and *p* = 0.009, respectively). It showed no significant differences in abundance of microbiota to treatment of chemotherapy and radiotherapy before surgery (data not shown).

### Association between microbial abundance and prognosis

To investigate whether the individual relative abundance of microbiota was associated with prognosis, we performed univariate and/or multivariate Cox regression analysis for overall survival (Table 3). The relative abundance of *Abiotrophia*, *Amaricoccus*, *Blastococcus*, *Bosea*, *Delftia*, *Dokdonella*, *Flavobacterium*, *Gemella*, *Granulicatella*, *Haemophilus*, *Leucobacter*, *Pelagibacterium*, *Sphingopyxis*, *Streptococcus* and *Williamsia* were significantly correlated with prognosis on univariate and multivariate Cox regression analysis after adjustment for clinicopathologic variables, such as ASA score, age, sex, and preoperative chemotherapy (Table 3-1). In the bile duct lesions, the relative abundance of *Delftia*, *Dermacoccus*, *Haemophilus*, *Leucobacter*, *Methylocapsa* and *Staphylococcus* was significantly associated with prognosis on univariate and multivariate Cox regression analysis after adjustment for clinicopathologic variables, such as ASA score and the presence of lymph node metastasis (Table 3-2). In the pancreas lesion, the relative abundance of *Abiotrophia*, *Aureimonas*, *Flavobacterium*, *Gemella*, *Howardella*, *Klebsiella*, *Proteus*, *Pelagibacterium*, *Sphingopyxis* and *Williamsia* showed a significant correlation with prognosis on univariate and multivariate Cox regression analysis after adjustment for clinicopathologic variables, such as ASA Score, Age, Sex, the presence of lymph node metastasis, and preoperative chemotherapy (Table 3-3). There is no common microbe correlate with prognosis between the bile duct lesion and the pancreatic lesion.

### Evaluation of threshold value of individual microbiota relative abundance for prognosis

We evaluated the threshold value of individual microbiota relative abundance for predicting prognosis using Cox proportional hazards model analysis (summarized in Table 4). In total samples, these groups with a high relative abundance of *Enterococcus*, *Eggerthella*, *Klebsiella*, *Corynebacterium*, *Moraxella*, *Hungatella*, *Paracoccus*, *Dermacoccus*, *Citrobacter*, *Lawsonella* and *Pseudoxanthomonas* showed a significantly poor prognosis compared with the other group (HR = 1.65, HR = 2.22, HR = 2.21, HR = 2.36, HR = 2.27, HR = 2.74, HR = 2.50, HR = 3.14, HR = 2.60, HR = 3.48 and HR = 7.41, respectively). On the other hand, these groups with a high relative abundance of *Streptococcus*, *Escherichia*, *Veillonella* and *Dialister* showed a significantly better prognosis compared with the other group (HR = 0.60, HR = 0.59, HR = 0.50 and HR = 0.35, respectively). In *Eggerthella* and *Corynebacterium*, the Fisher exact test revealed that there was significant difference in the Sex between these two groups divided by these threshold values (*p* = 0.026 and *p* = 0.046, respectively. Supplementary Table 3).

In the bile duct lesions, the group with high relative abundance of *Enterococcus*, *Corynebacterium*, *Haemophilus*, *Lawsonella*, *Staphylococcus*, *Moraxella*, *Bacteroides* and *Hungatella* showed a significantly poor prognosis compared with the other group (HR = 1.13, HR = 3.32, HR = 4.88, HR = 4.42, HR = 2.81, HR = 3.65, HR = 3.88 and HR = 5.42, respectively). On the other hand, these groups with a high relative abundance of *Streptococcus*, *Fusobacterium* and *Veillonella* showed a significantly better prognosis compared with the other group (HR = 0.35, HR = 0.26 and HR = 0.20, respectively). In *Staphylococcus* and *Veillonella*, the Fisher exact test revealed that there was significant difference in the Age between these two groups divided by these threshold values (*p* = 0.021 and *p* = 0.003, respectively. Supplementary Table 3).

In the pancreatic lesions, the group with high relative abundance of *Klebsiella*, *Veillonella*, *Acinetobacter*, *Selenomonas* and *Paracoccus* showed a significantly poor prognosis compared with the other group (HR = 1.82, HR = 2.87, HR = 2.88, HR = 3.49 and HR = 4.04, respectively). On the other hand, these groups with a high relative abundance of *Enterococcus*, *Staphylococcus*, *Bacteroides*, *Raoultella* and Streptococcus showed a significantly better prognosis compared with the other group (HR = 0.37, HR = 0.28, HR = 0.37, HR = 0.12 and HR = 0.55, respectively). The Fisher exact test revealed that there was significant difference in the ASA score between these two groups divided by these threshold values in *Raoultella*.

## Discussion

Pancreaticobiliary tract cancers, such as PDAC, cholangiocarcinoma, and gallbladder carcinoma, are aggressive malignancies with a high risk of invasion and metastasis. Furthermore, they are resistant to most cytotoxic agents [4, 5, 22, 23]. Due to the lack of sensitive clinical methods to detect these pancreaticobiliary tract cancers, most chemotherapeutic patients are often diagnosed at advanced stages and show an abysmal prognosis [24–26]. Thus, it is critical to establish new diagnostic, prognostic, and therapeutic biomarkers. Recent studies of the microbiota in the colorectum have suggested numerous links between these microbial communities and colorectal cancers [13, 16]. However, the association of changes in the microbiota in the gallbladder with pancreaticobiliary tract and gallbladder cancer has been rarely reported. In this study, we find a significant association between the relative abundance of certain microbes in gallbladder and the malignancy of lesion. Then, these microbes showed association with good prognosis in cholangiocarcinoma, but with poor prognosis in pancreatic adenocarcinoma, and vice versa.

The microbiota in the normal gallbladder consists of five main phyla: *Proteobacteria*, *Firmicutes*, *Bacteroidetes*, *Fusobacteria*, and *Actinobacteria* [18]. We also found an equivalent relative abundance of phyla in the bile juice derived from gallbladders in our study with pancreaticobiliary and gallbladder lesions. NMDS and PCoA analysis did not show distinct clustering, indicating the absence of overall microbiome differences among the types and locations of lesions. However, there was a significant association between trends in these variances and the N-score of the TNM staging system. Moreover, clustering analysis using relative abundance showed 2 cluster have significantly different prognosis in bile duct lesion. These results suggested that microbiota in gallbladder showed association with pancreaticobiliary and gallbladder cancer malignancy. In the cholangiocarcinoma, genes relative abundance analysis and clinical information showed that *Canpylobactor*, *Citrobacter* and *Leptotrichia* increased as the progress of lymphonode metastasis. Similarly, in the pancreatic adenocarcinoma, *Phyllobacterium* and *Sphingomonas* increased as the progress of lymphonode metastasis. In contrast, *Enterobacter*, *Hungatella* and *Mycolicibacterium* decreased. Our results support these findings of a wide range of infectious etiologies caused by *Campylobactor*, *Leptotrichia*, *Phyllobacterium* and *Sphingomonas* at different anatomic sites have been reported in the literature, suggesting its highly pathogenic potential [27–29]. Collectively, these results suggest that the progress of pancreaticobiliary tract cancer may affect changes in the gallbladder environment and, in turn, microbiome composition. In particularly, *Schaalia*, *Alloprevotella*, *Bilophila*, *Dialister*, *Eggerthella*, *Selenomonas* and *Streptococcus* showed a higher relative abundance in invasive intraductal papillary mucinous carcinoma than in intraductal papillary mucinous neoplasms. Thus, the pancreatic lesions affect the gallbladder microbiota, despite the pancreas being anatomically distant from the gallbladder.

The presence of *Delftia*, *Dermacoccus*, *Haemophilus*, *Leucobacter*, *Methylocapsa* and *Staphylococcus* were prognostic factors after adjustment for clinicopathologic variables in bile duct lesion. *Delftia*, *Haemophilus* and *Leucobacter* were common factor between the total cases and bile duct lesion cases. On the other hand, in pancreatic lesion analysis, *Abiotrophia*, *Aureimonas*, *Flavobacterium*, *Gemella*, *Howardella*, *Klebsiella*, *Proteus*, *Pelagibacterium*, *Sphingopyxis* and *Williamsia* were prognostic factors after adjustment for clinicopathologic variables. *Abiotrophia*, *Flavobacterium, Gemella, Pelagibacterium, Sphingopyxis* and *Williamsia* were common factor between the total cases and pancreatic lesion cases. Naito et.al., indicates that using human enteroids derived from the transverse colon, lipopolysaccharide from crypt-specific core microbiota (i.e., *Acinetobacter*, *Delftia*, and *Stenotrophomonas*) induced an increase in goblet cell-associated proteins such as MUC2 [30]. Unusual expression of mucin in pancreaticobiliary cancer may be responsible for mucosal microbiota. Genus-level analyses showed that four genera (*Actinomyces*, *Atopobium*, *Fusobacterium*, and *Haemophilus*) were present in significantly high proportions in colorectal cancer [31]. Tumor and the peri-tumoral regions of prostate had a higher relative abundance of Staphylococcus compared to normal areas [32]. There were some patients with cholangiocarcinoma and pancreatic adenocarcinoma that had a very high abundance of these genus, suggesting their direct involvement in malignant progression.

Furthermore, we found that the threshold value of relative abundance of microbiota is a significant marker for the prognosis of pancreaticobiliary tract cancer. These threshold values and clinical conditions, such as sex, age, American Society of Anesthesiologists (ASA) score, stages, and the administration of preoperative chemotherapy were non-confounding factors. In bile duct cancer, *Enterococcus*, *Staphylococcus* and *Bacteroides* were poor prognosis factors, but they were good prognosis factors for pancreatic cancer. *Streptococcus* was only common good prognosis factor. These results showed no common microbe correlate with poor prognosis between the bile duct lesion and the pancreatic lesion. Thus, the effect of the gallbladder environment was different for each lesion and that the difference in prognosis among the lesions might be caused by the gallbladder environment, including the effect of bile acid composition from each lesion or the distance of lesion from the gallbladder.

## Conclusions

In conclusion, this is the first report to indicate a link between gallbladder microbiota and pancreaticobiliary cancer prognosis. Although we took precautions in collecting biliary fluid from surgically resected gallbladders to prevent contamination by gastric or duodenal juices, it is impossible to completely exclude the likelihood of contamination because fluid samples from the stomach, duodenum, and intestines were not cross-checked in this study. However, our findings demonstrate that the alterations in the gallbladder microbiota population could be used to accurately distinguish the overall survival prognosis in pancreaticobiliary tract cancer patients after surgery. Further studies of these microbial markers are necessary to facilitate patient counseling, decision making regarding individualized therapy, and follow-up scheduling.

## Methods

### Bile samples and patients

We obtained 244 bile juice samples from surgically resected gallbladders. These bile juice samples were obtained from the gallbladders by a pathologist using a needle and immediately frozen (− 70 °C). The clinicopathologic features of the 96 samples from the bile tract lesions, 105 samples from the pancreas lesions, 14 samples from the gallbladder lesions, and 12 samples from other lesions collected at Kagoshima University, Japan, from May 2009 to August 2018 are summarized in Table 1. The clinical features used in this study were TNM staging (tumor, T; nodes, N; and metastases, M), age, ASA physical status classification score, and preoperative chemotherapy. Almost all patients did not receive antibiotics prior to surgery. Therefore, we excluded the information about antibiotic treatment in the statistical analysis.

**Table 1:** Patient demographics and clinicopathologic characteristics of the 244 bile juice samples in our study

Age	mean	sd				
	68.5	11.8	(yo)			
Observation period	37.3	24.8	(month)			
ASA Score	2.1	0.9				
	Female	Male				
Sex (n)	90	154				
	positive	negative				
Chemotherapy (n)	46	195				
	BDAC	PDAC	GDAC	Panc. Cystic	benign	other
type (n)	99	77	12	27	15	14
	Bile Duct	Pancras	Gallbladder	other		
posision (n)	101	112	17	14		
		(n)		(n)		(n)
Panc Cystic lesion	IPMC	10	IPMN	20	NET	7
benign					(n)	
Bile duct	Chronic cholangitis				2	
Gallbladder	Chronic cholecystitis				3	
	Periampullary duodenal diverticulitis				1	
	gallstone				1	
Pancreas	Mucinous cystadenoma				1	
	Chronic pancreatitis				2	
	Serous cystadenoma				3	
	Solid-pseudopapillary neoplasm				2	
other	Diffuse large B cell lymphoma, GCB type				1	
	gastrointestinal stromal tumor				5	
	Low grade tublar adenoma				1	
	Metastasis of adenocarcinoma				2	
	Tubular adenocarcinoma				3	
	Tubular adenocarcinoma, well differentiated				2	

*SD* Standard deviation, *ASA* American Society of Anaesthesiologists, *BDAC* Cholangiocarcinoma, *PDAC* Pancreatic ductal adenocarcinoma, *GDAC* Gallbladder cancer, *Panc. cystic* Pancreas cystic lesion, *IPMC* Intraductal papillary mucinous carcinoma, *IPMN* Intraductal papillary mucinous neoplasm

**Table 2:** Comparison relative abundance (permyriad) of microbiota among clinical informations

1. Sex		Female		Male				
		mean	sd	mean	sd	*p* (Wilcox test)	Cliff’s delta	CI95
	*Escherichia.Shigella*	602.3	1176.4	465.6	1303.6	0.007	0.208	(0.06–0.34)
	*Streptococcus*	188.5	407.7	154.3	376.3	0.030	0.165	(0.02–0.30)
2. Age		≧70		< 70				
		mean	sd	mean	sd	*p* (Wilcox test)	Cliff’s delta	CI95
	*Sphingomonas*	2.0	6.3	1.1	5.2	0.011	0.113	(0.03–0.20)
	*Fusobacterium*	319.5	717.4	420.6	705.5	0.044	−0.144	(−0.28–0.00)
3. progress								
	a. in BDAC	Early		Advance				
		mean	sd	mean	sd	*p* (Wilcox test)	Cliff’s delta	CI95
	*Campylobacter*	8.3	36.9	32.5	86.7	0.025	−0.212	(− 0.40 - -0.01)
	*Citrobacter*	57.9	392.1	93.0	226.3	0.001	−0.307	(− 0.49 - -0.10)
	*Leptotrichia*	0.0	0.4	41.2	194.6	0.007	−0.148	(− 0.21 - -0.09)
	b. in PDAC	Early		Advance				
		mean	sd	mean	sd	*p* (Wilcox test)	Cliff’s delta	CI95
	*Enterobacter*	896.3	899.5	518.5	959.1	0.004	0.368	(0.13–0.56)
	*Hungatella*	97.0	526.4	5.9	38.5	0.007	0.232	(0.06–0.39)
	*Mycobacterium*	12.2	26.3	4.3	15.7	0.018	0.231	(0.03–0.41)
	*Phyllobacterium*	845.6	1307.8	1514.4	1560.3	0.023	−0.286	(−0.50 - -0.04)
	*Sphingomonas*	1.0	4.7	3.2	8.5	0.058	−0.164	(−0.32–0.00)
	c. in pancreas cystic lesion	IPMN		IPMC				
		mean	sd	mean	sd	*p* (Wilcox test)	Cliff’s delta	CI95
	*Actinomyces*	0.3	1.1	4.6	7.0	0.038	−0.335	(−0.59 - -0.02)
	*Alloprevotella*	0.0	0.0	2.8	6.0	0.047	−0.200	(− 0.31 - -0.08)
	*Bilophila*	0.0	0.0	35.3	75.9	0.047	−0.200	(−0.31 - -0.08)
	*Dialister*	0.0	0.0	4.6	10.1	0.047	−0.200	(− 0.31 - -0.08)
	*Eggerthella*	0.0	0.0	26.6	56.6	0.047	−0.200	(−0.31 - -0.08)
	*Selenomonas*	0.0	0.0	0.9	2.0	0.047	−0.200	(− 0.31 - -0.08)
	*Streptococcus*	58.5	132.8	551.3	959.4	0.009	−0.600	(−0.86 - -0.09)

Early, without Lymphnode metastasis; Advanced, with Lymphnode metastasis; *IPMN* Intraductal papillary mucinous neoplasm, *IPMC* Intraductal papillary mucinous carcinoma, *SD* Standard deviation, *BDAC* Cholangiocarcinoma, *PDAC* Pancreatic ductal adenocarcinoma

**Table 3:** Multivariate or Univariate Cox regression analysis each microbiota

1. Totall (*n* = 219, Death:69)*^a^	Univariate			Multivariate		
microbe	Z-score	*p* value		Z-score	*p* value	
*Abiotrophia*	2.180	0.029	*	2.757	0.006	**
*Amaricoccus*	4.735	<0.001	***	4.497	<0.001	***
*Blastococcus*	3.239	0.001	**	3.668	<0.001	***
*Bosea*	3.239	0.001	**	3.668	<0.001	***
*Delftia*	2.374	0.018	*	2.965	0.003	**
*Desulfovibrio*	2.016	0.044	*	1.139	0.255	
*Dokdonella*	3.239	0.001	**	3.668	<0.001	***
*Enterococcus*	2.299	0.022	*	1.087	0.277	
*Flavobacterium*	3.737	<0.001	***	3.608	<0.001	***
*Gemella*	2.290	0.022	*	3.169	0.002	**
*Granulicatella*	2.087	0.037	*	1.975	0.048	*
*Haemophilus*	2.805	0.005	**	2.480	0.013	*
*Lawsonella*	1.876	0.061		3.754	<0.001	***
*Leucobacter*	3.025	0.002	**	3.351	0.001	***
*Pelagibacterium*	3.418	0.001	***	2.563	0.010	*
*Robinsoniella*	2.054	0.040	*	1.150	0.250	
*Sphingopyxis*	3.418	0.001	***	2.563	0.010	*
*Staphylococcus*	0.253	0.801		2.645	0.008	**
*Streptococcus*	−2.138	0.033	*	−2.042	0.041	*
*Williamsia*	2.079	0.038	*	2.020	0.043	*
2. Bile Duct lesion (*n* = 92, Death:18)*^b^	Univariate			Multivariate		
microbe	Z-score	*p* value		Z-score	*p* value	
*Delftia*	2.896	0.004	**	2.580	0.010	**
*Dermacoccus*	2.612	0.009	**	2.363	0.018	*
*Haemophilus*	2.838	0.005	**	2.786	0.005	**
*Lawsonella*	1.473	0.141		2.549	0.011	*
*Leucobacter*	3.351	0.001	***	3.572	<0.001	***
*Methylorosula*	2.114	0.035	*	2.332	0.020	*
*Parabacteroides*	2.114	0.035	*	1.360	0.174	
*Raoultella*	1.194	0.232		2.138	0.032	*
*Robinsoniella*	2.114	0.035	*	1.360	0.174	
*Staphylococcus*	3.643	<0.001	***	3.458	0.001	***
3. Pancreas lesion (*n* = 100, Death:43)*^c^	Univariate			Multivariate		
microbe	Z-score	*p* value		Z-score	*p* value	
*Abiotrophia*	1.977	0.048	*	2.428	0.015	*
*Akkermansia*	0.124	0.902		2.032	0.042	*
*Alloprevotella*	2.162	0.031	*	1.641	0.101	
*Aureimonas*	2.852	0.004	**	2.125	0.034	*
*Citrobacter*	1.343	0.179		1.983	0.047	*
*Flavobacterium*	3.126	0.002	**	2.780	0.005	**
*Gemella*	2.148	0.032	*	3.074	0.002	**
*Granulicatella*	2.140	0.032	*	1.709	0.088	
*Howardella*	2.698	0.007	**	1.974	0.048	*
*Hungatella*	1.851	0.064		3.026	0.002	**
*Klebsiella*	2.690	0.007	**	2.484	0.013	*
*Lawsonella*	1.708	0.088		2.273	0.023	*
*Novosphingobium*	2.458	0.014	*	0.178	0.859	
*Ochrobactrum*	2.698	0.007	**	1.974	0.048	*
*Pelagibacterium*	2.852	0.004	**	2.125	0.034	*
*Pseudomonas*	−0.947	0.343		−2.269	0.023	*
*Sphingopyxis*	2.852	0.004	**	2.125	0.034	*
*Williamsia*	2.835	0.005	**	2.124	0.034	*

^a^Multivariate cox regression alaysis with ASA score, age, sex, and preoperative chemotherapy

^b^Multivariate cox regression alaysis with ASA score and the presence of lymph node metastasis

^c^Multivariate cox regression alaysis with ASA Score, Age, Sex, the presence of lymph node metastasis, and preoperative chemotherapy. * *p* ≤ 0.05, ** *p* ≤ 0.01, *** *p* ≤ 0.001

**Table 4:** Assosiation between prognosis and threshold value of relative abundance (permyriad)

1. Totall (*n* = 219, Death:69)					
	Threshold value	(n)	HR	Cl95	*p*
*Enterococcus*	≧2023	(106)	1.65	(1.02–2.68)	0.040
*Eggerthella*	≧1	(15)	2.22	(1.01–4.86)	0.043
*Klebsiella*	≧1442	(13)	2.21	(1.01–4.83)	0.043
*Corynebacterium*	≧47	(14)	2.36	(1.13–4.94)	0.019
*Enhydrobacter*	≧59	(11)	2.27	(1.04–4.96)	0.034
*Hungatella*	≧96	(9)	2.74	(1.10–6.83)	0.025
*Paracoccus*	≧3	(8)	2.50	(1.01–6.22)	0.041
*Dermacoccus*	≧6	(7)	3.14	(1.14–8.65)	0.020
*Citrobacter*	≧260	(7)	2.60	(1.04–6.49)	0.032
*Lawsonella*	≧18	(5)	3.48	(1.09–11.11)	0.026
*Pseudoxanthomonas*	≧1	(4)	7.41	(2.29–23.96)	<0.001
*Streptococcus*	≧14	(127)	0.60	(0.37–0.96)	0.031
*Escherichia.Shigella*	≧35	(114)	0.59	(0.37–0.95)	0.029
*Veillonella*	≧51	(55)	0.50	(0.26–0.96)	0.033
*Dialister*	≧1	(29)	0.35	(0.13–0.96)	0.032
2. Bile Duct lesion (*n* = 92, Death:18)					
	Threshold value	(n)	HR	Cl95	*p*
*Enterococcus*	≧581	(85)	1.13	(1.16–1.49)	0.049
*Corynebacterium*	≧14	(12)	3.32	(1.13–9.72)	0.041
*Haemophilus*	≧198	(6)	4.88	(1.35–17.63)	0.007
*Lawsonella*	≧1	(13)	4.42	(1.36–14.37)	0.007
*Staphylococcus*	≧9	(26)	2.81	(1.01–7.80)	0.040
*Enhydrobacter*	≧27	(8)	3.65	(1.02–13.10)	0.032
*Bacteroides*	≧897	(9)	3.88	(1.07–14.05)	0.026
*Hungatella*	≧96	(3)	5.42	(1.18–24.80)	0.017
*Streptococcus*	≧18	(52)	0.35	(0.12–1.01)	0.042
*Fusobacterium*	≧3	(46)	0.26	(0.07–0.92)	0.025
*Veillonella*	≧1	(40)	0.20	(0.04–0.87)	0.017
3. Pancreas lesion (*n* = 100, Death:43)					
	Threshold value	(n)	HR	Cl95	*p*
*Klebsiella*	≧15	(45)	1.82	(0.99–3.35)	0.049
*Veillonella*	≧334	(9)	2.87	(1.11–7.39)	0.022
*Acinetobacter*	≧85	(8)	2.88	(1.11–7.45)	0.024
*Selenomonas*	≧2	(5)	3.49	(1.06–11.52)	0.030
*Paracoccus*	≧3	(4)	4.04	(1.23–13.21)	0.013
*Enterococcus*	≧149	(105)	0.37	(0.14–0.95)	0.031
*Staphylococcus*	≧68	(22)	0.28	(0.10–0.79)	0.011
*Bacteroides*	≧477	(26)	0.37	(0.15–0.94)	0.030
*Raoultella*	≧210	(14)	0.12	(0.02–0.89)	0.013
*Streptococcus*	≧480	(8)	0.55	(0.46–0.66)	0.046

### DNA extraction from bile juice samples

DNA from bile juice was extracted using a QIAamp PowerFecal DNA Kit (Qiagen, Valencia, CA). The quality and quantity of DNA were measured using the Qubit 3 system (Invitrogen, Carlsbad, CA). The bacterial 16S rRNA gene was amplified with Pro341F/Pro805R (V3–V4) primers [33] designed with Nextera overhang adapters (Illumina, San Diego, CA) using KAPA HiFi HotStart ReadyMix DNA polymerase (Roche Diagnostics Ltd., Burgess Hill, UK) to construct amplicon libraries. A second PCR step was performed to attach dual indices and Illumina sequencing adapters with Nextera XT index primers (Integrated DNA Technologies, Coralville, IA) using KAPA HiFi HotStart ReadyMix DNA polymerase (Roche Diagnostics). The resultant amplicons were purified using AMPure XP beads (Beckman Coulter, Brea, CA). After PCR products were quantified, equimolar ratios from each sample were pooled and sequenced on a MiSeq System (Illumina) according to the manufacturer’s instructions. It showed no amplification that the negative control using PBS buffer apply to similarly steps for DNA extraction and several PCR.

### Taxonomic assignment

Raw reads obtained from the sequencer were filtered according to the barcode and primer sequences using the MiSeq system. Then, the reads were imported into QIIME2 [34] v2019.4 in Linux, and quality assessment, filtering, and chimera detection were performed using the DADA2 pipeline. Taxonomic classification was assigned to amplicon sequence variants using 99% clustering in SILVA 132 (https://www.arb-silva.de/download/archive/) [35]. All samples were rarefied to the lowest reads, i.e., 10,000 reads, to minimize the effects of sequencing depth on alpha and beta diversity measures using “qiime feature-table rarefy” in Qiime2. The amplicon sequence variants were adequately detected for relative abundance (permyriad of total sequences) and alpha-diversity determination (i.e., Shannon Index, Cho1, Faith phylogenetic diversity [PD], and Pielou evenness index).

### Statistical analysis

Data were analyzed using the R computing environment v4.0.2 [36]. The normality of the data distribution was evaluated using the Kolmogorov–Smirnov test. Differences between groups were analyzed using the Welch *t*-test. A nonparametric test of group differences was performed using the Mann–Whitney *U* test (Wilcoxon rank sum test) and Kruskal-Wallis test. A parametric test of group differences was performed using the student t test. The effect size was calculated on cliff’s delta as nonparametrical and cohen’s d as parametrical. Categorical variables were compared by the Fisher exact test. Survival was estimated by the Kaplan–Meier method. The Cox proportional hazards model and survival curves were compared using the log-rank test. Prognostic factors were adjusted by a Cox regression model. All of prognosis analysis were performed on overall survival. The output data from QIIME2 were mined to determine alpha and beta diversity analyses and composition analysis in R using qiime2R, phyloseq, effesize and tidyverse packages. The clustering analysis on vegan package. A *p*-value < 0.05 was considered statistically significant.

## Supplementary Information

**Additional file 1: Supplementary Table 1.** The assignation of taxonomic lavel on main sequence of AVSs ugin BlastN.

**Additional file 2: Supplementary Table2.** Correlation analysis between clinical information and NMDS or PCoA.

**Additional file 3: Supplementary Table 3.** Cross-analysis between threshold value of relative abundance (permyriad) and clinical information.

## Data Availability

The datasets generated and/or analyzed during the current study is available from the corresponding author on reasonable request.

## References

[1] Khan SA, Thomas HC, Davidson BR, Taylor-Robinson SD (2005). Cholangiocarcinoma. Lancet.

[2] Kanthan R, Senger JL, Ahmed S, Kanthan SC (2015). Gallbladder Cancer in the 21st century. J Oncol.

[3] Gupta R, Amanam I, Chung V (2017). Current and future therapies for advanced pancreatic cancer. J Surg Oncol.

[4] Uesaka K, Boku N, Fukutomi A, Okamura Y, Konishi M, Matsumoto I (2016). Adjuvant chemotherapy of S-1 versus gemcitabine for resected pancreatic cancer: a phase 3, open-label, randomised, non-inferiority trial (JASPAC 01). Lancet..

[5] Valle J, Wasan H, Palmer DH, Cunningham D, Anthoney A, Maraveyas A (2010). Cisplatin plus gemcitabine versus gemcitabine for biliary tract cancer. N Engl J Med.

[6] Hundal R, Shaffer EA (2014). Gallbladder cancer: epidemiology and outcome. Clin Epidemiol.

[7] Hickman L, Contreras C (2019). Gallbladder Cancer: diagnosis, surgical management, and adjuvant therapies. Surg Clin North Am.

[8] Javle M, Zhao H, Abou-Alfa GK (2019). Systemic therapy for gallbladder cancer. Chin Clin Oncol.

[9] Qin J, Li R, Raes J, Arumugam M, Burgdorf KS, Manichanh C (2010). A human gut microbial gene catalogue established by metagenomic sequencing. Nature..

[10] Sender R, Fuchs S, Milo R (2016). Revised estimates for the number of human and Bacteria cells in the body. PLoS Biol.

[11] Human microbiome project C. a framework for human microbiome research. Nature. 2012;486(7402):215–21. 10.1038/nature11209. 10.1038/nature11209 PMC3377744 22699610

[12] Blum HE (2017). The human microbiome. Adv Med Sci.

[13] Kamada N, Seo SU, Chen GY, Nunez G (2013). Role of the gut microbiota in immunity and inflammatory disease. Nat Rev Immunol.

[14] Gilbert JA, Blaser MJ, Caporaso JG, Jansson JK, Lynch SV, Knight R (2018). Current understanding of the human microbiome. Nat Med.

[15] Zitvogel L, Ma Y, Raoult D, Kroemer G, Gajewski TF (2018). The microbiome in cancer immunotherapy: diagnostic tools and therapeutic strategies. Science..

[16] Kitamoto S, Nagao-Kitamoto H, Jiao Y, Gillilland MG, Hayashi A, Imai J (2020). The Intermucosal connection between the mouth and gut in commensal Pathobiont-driven colitis. Cell..

[17] Serra N, Di Carlo P, D'Arpa F, Battaglia E, Fasciana T, Gulotta G (2021). Human bile microbiota: a retrospective study focusing on age and gender. J Infect Public Health.

[18] Molinero N, Ruiz L, Milani C, Gutierrez-Diaz I, Sanchez B, Mangifesta M (2019). The human gallbladder microbiome is related to the physiological state and the biliary metabolic profile. Microbiome..

[19] Chng KR, Chan SH, Ng AHQ, Li C, Jusakul A, Bertrand D (2016). Tissue microbiome profiling identifies an enrichment of specific enteric Bacteria in Opisthorchis viverrini associated Cholangiocarcinoma. EBioMedicine..

[20] Geller LT, Barzily-Rokni M, Danino T, Jonas OH, Shental N, Nejman D (2017). Potential role of intratumor bacteria in mediating tumor resistance to the chemotherapeutic drug gemcitabine. Science..

[21] Altschul SF, Gish W, Miller W, Myers EW, Lipman DJ (1990). Basic local alignment search tool. J Mol Biol.

[22] Adamska A, Domenichini A, Falasca M. Pancreatic ductal adenocarcinoma: current and evolving therapies. Int J Mol Sci. 2017;18(7). 10.3390/ijms18071338. 10.3390/ijms18071338 PMC5535831 28640192

[23] Egawa S, Toma H, Ohigashi H, Okusaka T, Nakao A, Hatori T (2012). Japan pancreatic Cancer registry; 30th year anniversary: Japan pancreas society. Pancreas..

[24] Singhi AD, Koay EJ, Chari ST, Maitra A (2019). Early detection of pancreatic Cancer: opportunities and challenges. Gastroenterology..

[25] Kaur S, Baine MJ, Jain M, Sasson AR, Batra SK (2012). Early diagnosis of pancreatic cancer: challenges and new developments. Biomark Med.

[26] Rizvi S, Khan SA, Hallemeier CL, Kelley RK, Gores GJ (2018). Cholangiocarcinoma - evolving concepts and therapeutic strategies. Nat Rev Clin Oncol.

[27] Shinha T (2015). Fatal bacteremia caused by Campylobacter gracilis, United States. Emerg Infect Dis.

[28] Hou H, Chen Z, Tian L, Sun Z (2018). Leptotrichia trevisanii bacteremia in a woman with systemic lupus erythematosus receiving high-dose chemotherapy. BMC Infect Dis.

[29] Zheng R, Wang G, Pang Z, Ran N, Gu Y, Guan X (2020). Liver cirrhosis contributes to the disorder of gut microbiota in patients with hepatocellular carcinoma. Cancer Med.

[30] Naito T, Mulet C, De Castro C, Molinaro A, Saffarian A, Nigro G, et al. Lipopolysaccharide from crypt-specific Core microbiota modulates the colonic epithelial proliferation-to-differentiation balance. mBio. 2017;8(5). 10.1128/mBio.01680-17. 10.1128/mBio.01680-17 PMC5646255 29042502

[31] Kasai C, Sugimoto K, Moritani I, Tanaka J, Oya Y, Inoue H (2016). Comparison of human gut microbiota in control subjects and patients with colorectal carcinoma in adenoma: terminal restriction fragment length polymorphism and next-generation sequencing analyses. Oncol Rep.

[32] Cavarretta I, Ferrarese R, Cazzaniga W, Saita D, Luciano R, Ceresola ER (2017). The microbiome of the prostate tumor microenvironment. Eur Urol.

[33] Takahashi S, Tomita J, Nishioka K, Hisada T, Nishijima M (2014). Development of a prokaryotic universal primer for simultaneous analysis of Bacteria and Archaea using next-generation sequencing. PLoS One.

[34] Caporaso JG, Kuczynski J, Stombaugh J, Bittinger K, Bushman FD, Costello EK (2010). QIIME allows analysis of high-throughput community sequencing data. Nat Methods.

[35] Quast C, Pruesse E, Yilmaz P, Gerken J, Schweer T, Yarza P (2013). The SILVA ribosomal RNA gene database project: improved data processing and web-based tools. Nucleic Acids Res.

[36] R Core Team. R: A language and environment for statistical computing. Vienna, Austria: R Foundation for Statistical Computing; 2016. https://www.R-project.org/.

